# Risk factors associated with chronic kidney disease progression: Long-term retrospective analysis from Qatar

**DOI:** 10.5339/qmj.2022.57

**Published:** 2022-11-30

**Authors:** Ahmed Farouk Hamdi, Ashraf Fawzy, Essa Abuhelaiqa, Muhammad Asim, Awais Nuaman, Adel Ashur, Omar Fituri, Mohamad Alkadi, Hassan Al-Malki

**Affiliations:** ^1^Hamad General Hospital, Hamad Medical Corporation, Qatar. Email and ORCID ID: ahamdi@hamad.qa & https://orcid.org/0000-0002-7289-4942

**Keywords:** chronic kidney disease, progression risk factors, end-stage kidney disease

## Abstract

Introduction: The risk factors influencing the natural course of chronic kidney disease (CKD) are complex and heterogeneous. Recognizing the factors associated with CKD progression can enable the identification of high-risk patients for more intensive treatment.

Patients and methods: A retrospective evaluation of CKD patients was performed under follow-up between January 1, 2001 and December 31, 2016 at a tertiary health care center.

Results: Among 5370 screened patients, 1020 patients with complete data were included in the analysis. The median follow-up period for the studied patients was 9.3 years. Based on the analysis, 120 (11.8%) patients had reached end-stage kidney disease “ESKD” or death. The study revealed that the risk factors associated with reaching ESKD and/or death using Kaplan–Meier survival curve and log rank test included higher hemoglobin A1c among diabetic patients, higher grade of proteinuria, and non use of renin-angiotensin system blockers. The patients with CKD progression constituted 77.2% of all CKD patients. The study findings indicated that older age, Arab ethnicity, smoking habit, diabetes mellitus and hypertension (presumed as original kidney diseases) are among the significant risk factors associated with a further decline of the estimated glomerular filtration rate (eGFR) and further CKD progression.

Conclusion: This study summarized the demographic and clinical risk factors associated with CKD progression and patients’ outcomes among a unique and heterogeneous population in the state of Qatar. Intensive treatment of modifiable risk factors could be of value in halting the progression of CKD. However, prospective studies are warranted to confirm our findings.

## Introduction

The global prevalence of chronic kidney disease (CKD) has been estimated by the Global Burden of Disease Study 2017 as 9.1% of the world population (697.5 million cases). Moreover, CKD resulted in 1.2 million deaths and was the 10^th^ leading cause of death worldwide in 2020.^1^

The risk factors influencing the natural course of CKD are complex and heterogeneous and there are only a few systematic studies that have focused on this issue.^2^ The estimated risk factors of CKD progression causing morbidity and mortality have been studied across different ethnic and racial populations, showing important differences between them.^3^ In the United States, for instance, the rate of renal replacement therapy (RRT) initiation for end-stage kidney disease (ESKD) is disproportionately higher for ethnic minority groups (such as African-American, Hispanic, and Native Americans) when compared to that for Caucasians, despite the similar prevalence for early stages of CKD.^4,5^

The economic development experienced in Qatar has been associated with inappropriate dietary and lifestyle patterns that led to increased rates of obesity and chronic non communicable diseases. Furthermore, this situation can be associated with a surge in international migration of workers from developing countries, as evidenced by the annual population growth of 18.9% from 2008 onward.^6^

The present work aimed to study the distribution of CKD in Qatar's heterogeneous population that has been followed in nephrology clinics to examine the prevalence of risk factors associated with CKD progression as well as to describe the relevant clinical outcomes (such as death or end-stage kidney disease) over a long observation period.

## Patients And Methods

A retrospective evaluation of CKD patients under follow-up at a tertiary health center (Hamad General Hospital) was conducted. In this study, we reviewed the electronic medical records of all patients visiting the nephrology clinics between January 1, 2001 and December 31, 2016. The estimated glomerular filtration rate (eGFR) for each patient was calculated from the serum creatinine level using the CKD-Epidemiology Collaboration equation.^7^ Patients diagnosed with CKD of different stages on at least 2 occasions and lasting >3 months were included. All patients with albuminuria (an albumin-to-creatinine ratio >3 mg/mmol) or equivalent (protein-to-creatinine ration >15 mg/mmol or urine protein reagent strip 1+ or more), on at least 2 occasions, lasting >3 months, were also included. All outpatient eGFR measurements until the end of the study were utilized, excluding the laboratory measurements associated with any hospital admission.

The identified CKD patients were screened for demographic data, including their age, gender, nationality, and ethnicity. Body mass index (BMI) was calculated for each patient as the body weight measured in kilograms, divided by the height squared meter. The date of diagnosis of CKD and eGFR at the time of diagnosis was recorded. Follow-up renal function until the last follow-up visit was performed yearly, and it included yearly registered values of eGFR for any individual patient plus the degree of albuminuria/proteinuria (expressed as urinary albumin or protein/creatinine ratios in mg/mmol). Further categorization of the eGFR and albuminuria grades for each value/year/patient was conducted as per the Clinical Practice Guideline for the Evaluation and Management of Chronic Kidney Disease (KDIGO) classification.^8^

The cause of CKD for each patient was retrieved from the patient's file as per the primary nephrologist assessment. In addition, the comorbid conditions, including diabetes mellitus, diabetes complications, hypertension, dyslipidemia, cardiovascular diseases, cerebrovascular disease, or peripheral vascular disease, were also assessed.

### Inclusion criteria

– Age ≥ 18 years at the time of inclusion.
– All patients diagnosed with CKD according to the KDIGO classification.
– Follow-up period with available laboratory data for ≥ 2 years.

### Exclusion criteria

– Follow-up period < 2 years.
– Patients who required initiation of RRT shortly after the presentation.
– Insufficient data, including:
• unidentified cause of CKD
• unavailable renal function test data for >2 years during the follow-up

### Primary outcomes

#### Included

– Initiation of RRT in the form of hemodialysis, peritoneal dialysis, or preemptive kidney transplantation
– Patient's death

### Secondary outcome

– eGFR progression:Patients were classified as per their eGFR difference between the values at the time of inclusion and the last follow-up, as follows: patients with eGFR decline (CKD progression patients, who show reduced eGFR values over years) and patients without (non progression patients who did not show reduced eGFR values over years).
– eGFR decline expressed in mL/min/per year for those with CKD progression:eGFR decline per year was calculated by dividing the eGFR difference (at the time of inclusion and the last follow-up) by the follow-up period in years.
– Albuminuria or proteinuria progression

### Statistics

Data were presented as the means ± standard deviation for continuous variables and as the numbers or percentages for categorical variables. Patients were followed in survival analysis from the date of CKD diagnosis until the last follow-up or the occurrence of primary outcome events, including the start of RRT, kidney transplant, eGFR < 10 mL/min/1.73 m^2^, or death. Kaplan–Meier analysis with the log rank test was used to assess the association of primary outcome events with different measured variables. *p* ≤ 0.05 was considered to indicate statistical significance.

## Results

**Figure 1.**
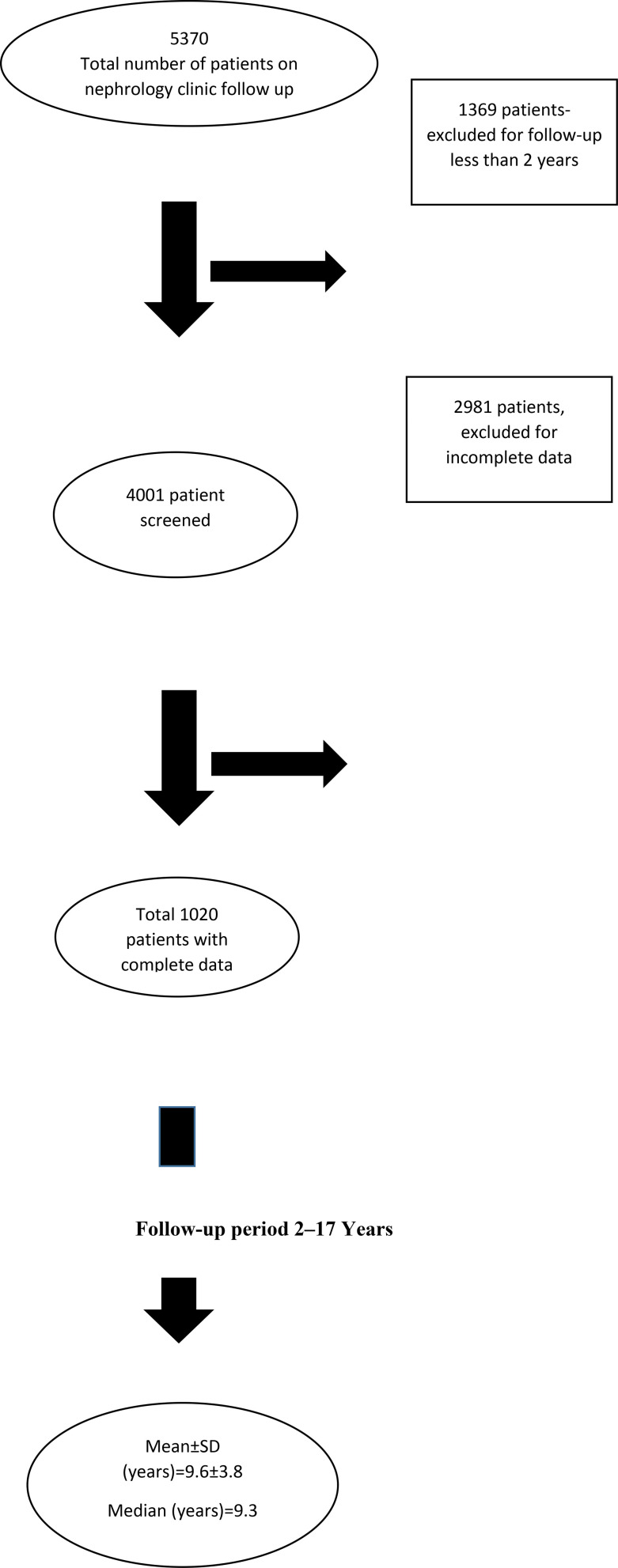
Study population flowchart and the follow-up period

In total, 5370 patients visited the nephrology outpatient department at a tertiary health care center between January 1, 2001 and December 31, 2016. A total of 1369 patients were excluded because they had < 2-year of the follow-up period. Further 2981 patients were excluded for incomplete data. The remaining 1020 patients with complete data were included in the analysis. The mean ± SD and median follow-up periods for the studied patients were 9.6 ± 3.8 and 9.3 years, respectively (range: 2–17 years) (Figure 1).

Table 1 shows the demographic characteristics of the patients. The patients mainly included men (63.6%) of Arab ethnicity (61.3%) and aged >60 years (55%). Their BMI, class I, II, and III constituted 46.7%. Table 1 also shows CKD, eGFR, and albuminuria grade distribution at the start and progression at the last follow-up. For example, G3b, G4, and G5 constituted 19.9 % at the start, which increased to 58.5% at the last follow-up. Similarly, the A3 albuminuria grade progressed from 36.9% at the start to 46.6% at the last follow-up.

Table 2 shows the cause of CKD, which was mainly secondary to DM (18.8%), HTN (15.9%), or both (41.5%). Glomerulonephritis constituted the next most frequent cause (9.2%). Evaluation of the various comorbid conditions associated with CKD revealed that hypertension was the most prevalent disease, detected in 93.5% of the studied patients (57.4% of which were diagnosed as a cause of CKD either alone or in association with DM). The majority of hypertensive patients (69.4%) were maintained on two or more anti-hypertensive medications. The prevalence of DM was 67.2% (60.3% of which were diagnosed as a cause of CKD either alone or in association with HTN). Other comorbidities are described in Table 2.

One hundred and twenty (11.8%) patients reached the primary outcome (ESKD or death) after a mean follow-up of 9.6 years (Table 2).

Table 3 describes the factors that were found to be associated with the decline of eGFR over the studied follow-up period when compared with those who did not experience any reduction of eGFR during the same period. Age >60 years, Arab ethnicity, and having a smoking habit were identified as the risk factors of CKD progression leading to morbidity and mortality (*p* < 0.05). Furthermore, DM and/or HTN, as a cause of CKD, was significantly associated with eGFR decline, when compared to other causes (*p* = 0.0001). For DM, higher hemoglobin A1c acted as a significant risk factor for eGFR decline, while, for HTN, the more the number of anti-hypertensive medications, the greater the ratio of eGFR decline. For all CKD patients, higher albuminuria grade and non use of ACEI/ARBs were significantly associated with more patients experiencing eGFR decline. All associated co morbidities were coupled with further eGFR decline, except for cerebrovascular accidents, which did not reach any statistical significance.

For patients who experienced eGFR decline, further analysis was performed. Table 4 shows the degree of eGFR decline as per the studied risk factors. Among the causes of CKD, having both DM and HTN as the primary cause of CKD was associated with the highest degree of eGFR decline (3.2 mL/min/1.73 m^2^/year) when compared to others. Furthermore, a higher degree of albuminuria, non use of ACEI/ARBs, and higher hemoglobin A1c level were associated with greater eGFR decline, reaching 3.2, 3.3, and 4.1 mL/min/1.73 m^2^/year, respectively.

**Figure 2.**
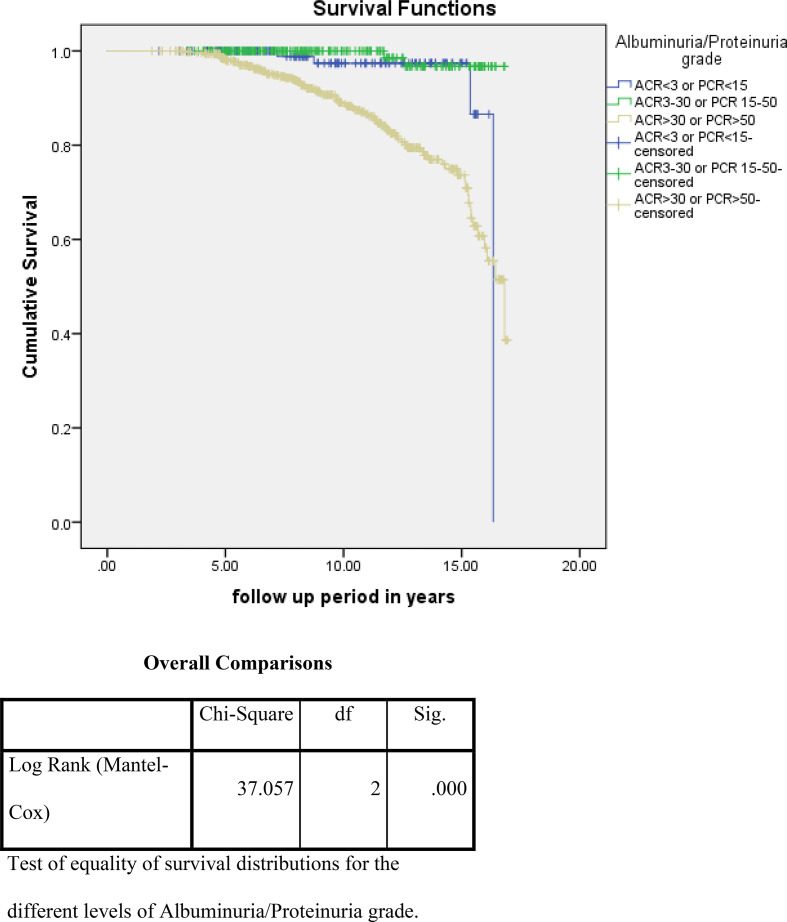
Impact of albuminuria grade on the primary outcome events (ESKD and death)

**Figure 3.**
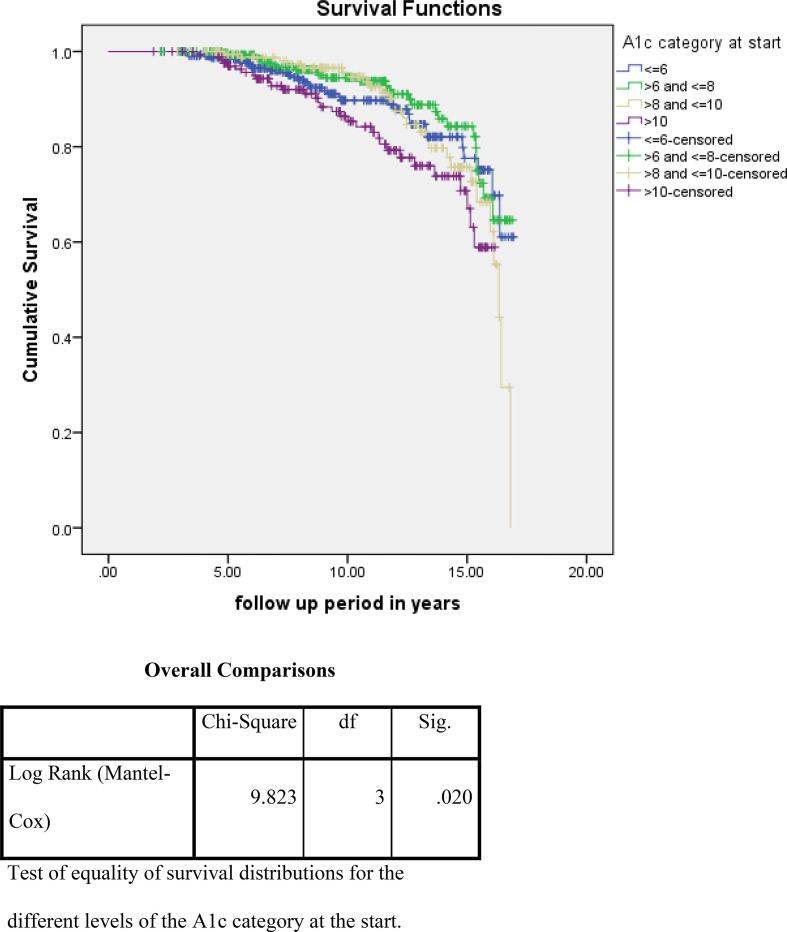
Impact of hemoglobin A1c at the time of diagnosis of CKD on the primary outcome events (ESKD and death)

**Figure 4.**
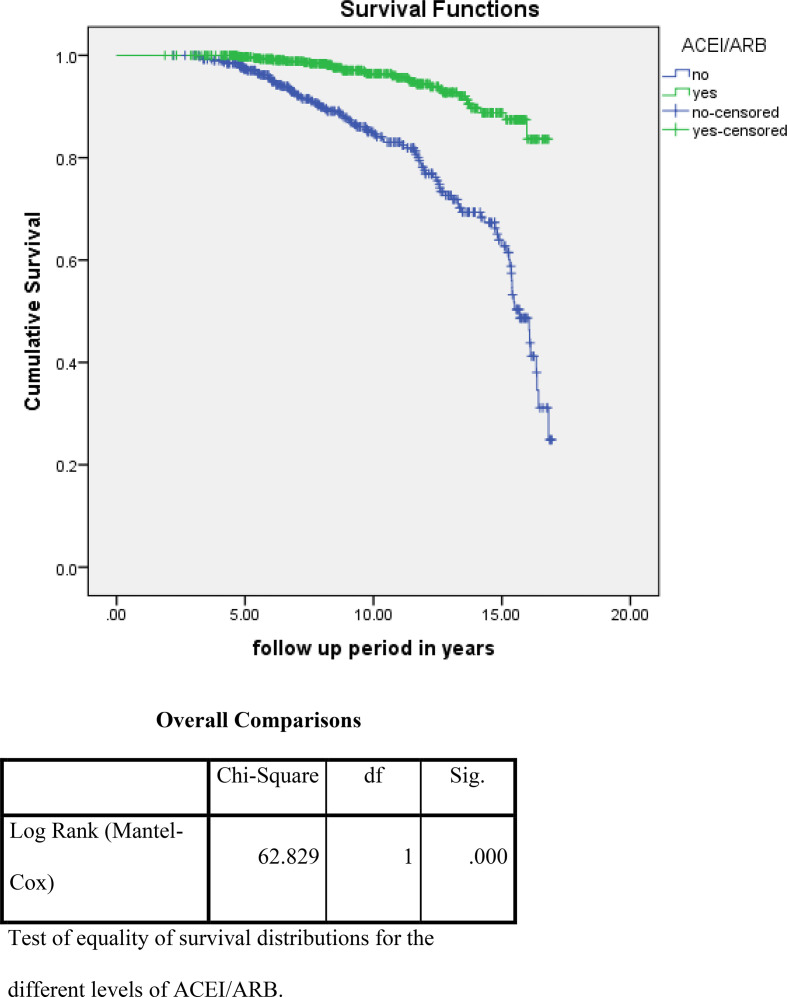
Impact of the use of ACEI/ARBs on the primary outcome events (ESKD and death)

For the analysis of risk factors associated with ESKD or death using the Kaplan–Meier survival curve and log rank test, we noted that higher albuminuria, higher A1c, and non use of ACE/ARBs were associated with a statistically high significance and increased incidence of these outcome events (Figures 2–4). On the other hand, for the analysis of the cause of CKD, having DM and HTN was associated with more ESKD and death; nevertheless, it did not reach statistical significance (5).

## Discussion

In this study, a long follow-up period associated with informative electronic medical files allowed the investigators to identify the clinical risk factors associated with CKD progression and those associated with ESKD/patient death over a prolonged period when compared to other similar studies. Among our studied group of CKD patients, CKD progression was found to be associated with patients’ certain demographics and disease-related risk factors.

Patient-related factors in our study included older age (particularly >60 years), Arab ethnicity, or being a smoker. Older age is associated with the occurrence and higher progression of CKD by multiple studies.^9,10^ Regarding the Arab racial preponderance, although non communicable diseases including diabetes, hypertension, and obesity are highly prevalent in the Arab world, there is very limited data available on the exact prevalence of various kidney diseases in the Arab population.^11^ One recent report from Palestine showed that CKD is highly prevalent among type 2 diabetic adults and that comorbid hypertension, smoking, and older age increase the risk of developing CKD.^12^ For patients with CKD, there has been a lack of studies on the association between smoking and CKD progression, and the limited literature has yielded conflicting results. However, in a recent prospective cohort study involving Korean CKD patients, smoking was found to be associated with a significantly higher risk of worsening kidney function.^13^

In a systematic review and meta-analysis with a follow-up duration of 1.5–16 years, it was found that being male, having substantial proteinuria, and having diabetes were associated with increased hazards of progression to ESRD.^14^ In our study, there was no gender effect on CKD progression. Some previous studies have indicated no gender differences in the progression of nephropathy among patients with diabetes.^15^ Other studies have shown that the male gender predicted the progression of renal disease in patients with diabetes,^16^ while others reported a detrimental effect of the female gender on the progression of diabetic nephropathy.^17^

Disease-related factors, associated with CKD progression in the current study, included having type 2 diabetes and hypertension (diagnosed as the causes of original kidney disease), diabetic retinopathy, diabetic neuropathy, diabetic foot, cardiovascular disease, or advanced CKD at the time of diagnosis. Furthermore, diabetes mellitus was found to be associated with greater CKD progression when associated with a higher Hb A1C. Intensive glucose control has previously been demonstrated to be associated with a significant reduction in renal events in patients with type 2 diabetes in the Action in Diabetes and Vascular Disease study.^18^ However, in contrast to our findings, the present finding mainly suggested the development of micro or macro albuminuria, with no effect on doubling the serum creatinine level. Other relatively small-sized studies reported no association between the baseline HbA1c level and renal outcomes.^19^ Therefore, a relatively long-term follow-up of our patients might have shown the long-term effect of Hb A1C in association with rising serum creatinine levels.

In our study, hypertension was found to be associated with greater CKD progression when the patient was treated with more numbers of anti-hypertensive medication (reflecting more severe hypertension) and if antihypertensives were not including ACEIs or ARBs. This finding is supported by several studies demonstrating the beneficial effects of blood pressure control and other benefits with the use of ACEs/Arbs in reducing renal events.^20,21^

The association between kidney disease progression and cardiovascular disease (CVD) risk was assessed among patients with type 2 diabetes and CKD in a UK primary healthcare setting to reveal that progression of CKD is possibly associated with CVD risk, in which increased risk of cardiovascular events was noted among those with the fastest rate of eGFR decline, most predominantly in heart failure cases.^22^ In our study, CKD progression was associated with CVDs including congestive heart failure, coronary artery disease, or peripheral vascular disease (but not cerebrovascular accidents).

Advanced CKD presentation including higher albuminuria stage and higher creatinine level was associated with more CKD progression. The presence of proteinuria, the major marker of kidney damage, is the best-known risk factor for CKD progression. Several studies have recorded observed that proteinuria was associated with a faster rate of kidney function decline and the achievement of ESRD.^23^

In our study, greater eGFR decline was found to be associated with having both diabetes and hypertension ( − 3.2 ± 4.3 mL/min/1.73 m^2^/year) as original kidney disease, to have a higher grade of albuminuria ( − 3.2 ± 2.4 mL/min/1.73 m^2^/year), high HbA1c (>10% was associated with an eGFR decline of − 4.1 ± 6.2 mL/min/1.73 m^2^/year) and not being on any medication for renin-angiotensin system block ( − 3.3 ± 3.7 mL/min/1.73 m^2^/year).

Similarly, on assessing factors associated with reaching primary outcome events, including death and reaching ESKD, it was found that higher albuminuria, high HbA1c, and the non use of renin-angiotensin system blockers were associated with a high statistical significance. On the other hand, having type 2 diabetes and hypertension as the primary causes of CKD were found to be associated with higher death/ESRD, albeit without significance.

### Limitations and strengths

Limitations included that this study was observational and hence its results cannot be used to suppose causality. In addition, being in a country with such a diverse population, the studied group was heterogonous. However, there are many strengths of this study. For instance, it included a patient selection process, which resulted in a representative CKD patient cohort as well as a long observation period with a high number of follow-up visits, which allowed the identification of risk factors for kidney and patient outcomes.

## Conclusions

Our findings showed that the risk factors associated with reaching ESKD and/or death included high A1c among diabetic patients, a high grade of proteinuria, and the non use of renin-angiotensin system blockers. Furthermore, the study revealed that older age, Arab ethnicity, smoking habit, diabetes mellitus and hypertension (presumed as original kidney diseases) are among the significant risk factors associated with greater eGFR decline and CKD progression.

### Financial Disclosure

Supported by a grant from Medical Research Center, #17195/17, Hamad Medical Corporation, Qatar

### Ethical Approval and Patients’ Consent

The study was approved by the local ethical committee of the Hamad Medical Corporation research center, under research study number #17195/17 in accordance with the relevant guidelines and regulations. Patients’ consent was inapplicable as the study was designed to be a retrospective evaluation of preexisting medical data from registries and electronic medical reports with no human interventions. Patients` confidentiality was preserved.

### Availability of Data and Materials

Available

### Competing Interests

None to disclose

### Authors’ Contributions

Contributing authors have shared in data collection and manuscript review.

### Acknowledgments

The authors acknowledge HMC medical research center, nephrology ward staff, and coordinators for their assistance.

## Figures and Tables

**Figure 5.**
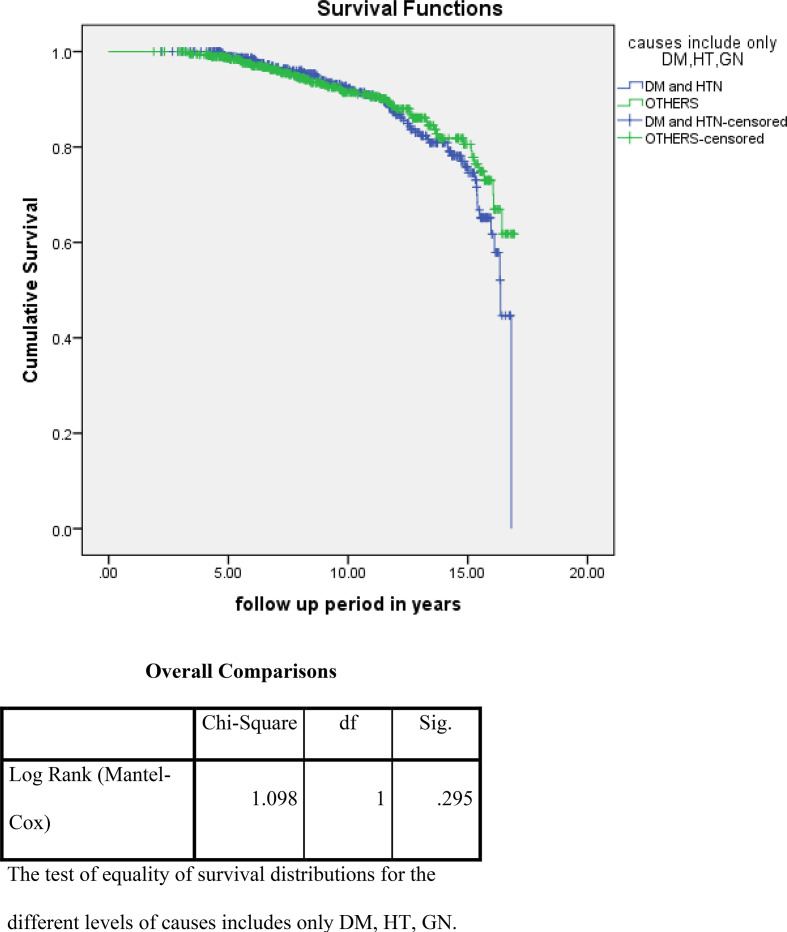
Impact of the cause of CKD on the primary outcome events (ESKD and death)

**Table 1:** Demographic characteristics of CKD patients

Age (years):	
Mean ± SD	59.8 ± 14.2
Median	61.3
More than 60 years	561 (55%)
Equal to or less than 60 years	459 (45%)
**Gender:**	
Males	649 (63.6%)
Females	371 (36.4%)
**Ethnicity:**	
Arab	625 (61.3%)
Asian (East, South East)	336 (32.9%)
Indian	28 (2.7%)
African	26 (2.5%)
Caucasian	5 (0.5%)
**BMI (kg/m^2^):**	
18.5 to < 25 (normal weight)	132 (12.9%)
≤ 25 to < 30 (over weight)	346 (33.9%)
30 to < 35 (class I obesity)	260 (25.4%)
35 to < 40 (class II obesity)	128 (12.5%)
≤ 40 (class III obesity)	90 (8.8%)
Missing	64 (6.2%)
Smoking	
Yes	188 (18.4%)
No	710 (69.6%)
Missing	122 (11.9%)
**CKD, at time of diagnosis:**	
1-Grades:	111 (10.9%)
G1	462 (41.8%)
G2	279 (27.4%)
G3a	147 (14.4%)
G3b	48 (4.7%)
G4	8 (0.8%)
G5	
2-Albuminuria:	241 (23.6%)
A1	386 (37.8%)
A2	376 (36.9%)
A3	17 (1.7%)
Missing	
**CKD, at last follow-up:**	
1-Grades:	
G1	60 (5.9%)
G2	169 (16.6%)
G3a	180 (17.6%)
G3b	238 (23.3%)
G4	214 (21%)
G5	145 (14.2%)
Missing	14 (1.4%)
2-Albuminuria:	
A1	135 (13.2%)
A2	227 (22.3%)
A3	475 (46.6%)
Missing	183 (17.9%)

**Table 2:** Causes of CKD, associated comorbidities, follow-up period, and outcome

Cause of CKD:	Number (%)
DM and HTN	424 (41.5%)
DM	192 (18.8%)
HTN	163 (15.9%)
GN	94 (9.2%)
Autoimmune	23 (2.2%)
Neoplasia	15 (1.4%)
Obstructive	8 (0.7%)
Solitary functioning kidney (dysplastic/surgical removal)	7 (0.6%)
Hereditary	2 (0.19%)
Chronic interstitial nephritis	2 (0.19%)
Acquired cystic kidney disease	1 (0.09%)
Cardio renal	1 (0.09%)
Atrophic kidneys	88 (8.6%)
**Co morbidities:**	**Number (%)**
HTN	954 (93.5%)
Number of Anti-hypertensive medications:	
1	292 (30.6%)
2	378 (39.6%)
3	216 (22.6%)
4	66 (6.9%)
5	2 (0.2%)
DM	686 (67.2%)
Hemoglobin A1c %:	
< = 6	55 (8%)
>6 and < = 8	400 (58.3%)
>8 and < = 10	174 (25.3%)
>10	57 (8.3%)
Diabetic Retinopathy	341 (33.4%)
Diabetic Neuropathy	183 (17.9%)
Diabetic foot	109 (10.6%)
Coronary artery disease	281 (27.5%)
Congestive heart failure	182 (17.8%)
Peripheral artery disease	91 (8.9%)
Cerebrovascular Stroke	65 (6.3%)
HCV	28 (2.7%)
HBV	11 (1%)
HIV	1 (0.09%)
**Follow-up period (years):**	
Mean ± SD	9.6 ± 3.8
Median	9.3
**Outcome:**	**Number (%)**
living on regular follow-up	900 (88.2%)
Expired	20 (2%)
Hemodialysis	73 (7.2%)
Peritoneal dialysis	24 (2.4%)
kidney transplant	3 (0.3%)

DM: diabetes mellitus

HTN: hypertension

GN: glomerulonephritis

HCV: hepatitis C virus infection

HBV: hepatitis B virus infection

HIV: Human immunodeficiency virus infection

**Table 3:** Factors affecting eGFR decline among the studied patients

Estimated GFR	Decliners	Non decliners	P value
Total = 1020	Number = 788 (77.2%)	Number = 232 (22.7%)	
**Gender:**			
Male	502 (77.3%)	147(22.7%)	0.491
Female	286 (77.1%)	85 (22.9%)	
**Age (years), last follow-up**		
Mean ± SD	61.8 ± 13	52.9 ± 16	0.0001*
More than 60 years	471 (83.9%)	90 (16.1%)	
Equal or less than 60 years	317 (69.1%)	142 (30.9%)	0.0001*
**Ethnicity:**			
Arab	504 (80.6%)	121 (19.4%)	
Others	284 (71.8%)	111 (28.2%)	0.001*
**BMI:**			
BMI at study entry	31.7 ± 6.0	29.7 ± 5.4	0.176
BMI at last follow-up	31.0 ± 6.8	30.2 ± 6.0	0.161
**Smoking:**			
Yes	160 (85.1%)	28 (14.9%)	
No	533 (75.1%)	177 (24.9%)	0.013*
**Cause of CKD:**			
DM and HTN	366 (86.1%)	59 (13.9%)	
DM	162 (84.4%)	29 (15.2%)	
HTN	116 (71.2%)	47 (28.8%)	
Others	144 (59.8%)	97 (40.2%)	0.0001*
**Albuminuria, at time of diagnosis:**			
Mean ± SD	27.3 ± 53.1	14.6 ± 23.1	0.015*
**Grade:**			
A1	166 (68.9%)	75 (31.1%)	
A2	308 (79.8%)	78 (20.2%)	
A3	302 (80.3%)	74 (19.7%)	0.001*
**Hemoglobin A1c:**			
At time of diagnosis of CKD	7.8 ± 2.3	6.7 ± 2.0	0.0001*
At last follow-up	7.1 ± 1.8	6.4 ± 1.5	0.0001*
Duration of hypertension, years	12.6 ± 7.7	10.3 ± 6.5	0.14
Number of anti-hypertensive medications			
1	207 (70.9%)	85 (29.1%)	
2	299 (79.1%)	79 (20.9%)	
3	178 (82.4%)	38 (17.6%)	0.0001*
4	63 (95.5%)	3 (4.5%)	
Use of ACEI/ARBs			
Yes	458 (74.1%)	166 (25.9%)	
No	330 (82.1%)	72 (17.9%)	0.002*
**Co morbidities:**			
Diabetes Mellitus			
YES	576 (84%)	110 (16%)	
No	212 (63.7%)	121 (36.3%)	0.0001*
Diabetic retinopathy			
Yes	303 (88.9%)	38 (11.1%)	
No	485 (71.4%)	194 (28.6%)	0.0001*
Diabetic Neuropathy			
Yes	169 (92.3%)	14 (7.7%)	
No	619 (14 %)	218 (26%)	0.0001*
Diabetic foot			
Yes	97 (88%)	12 (11%)	
No	691 (75.9%)	220 (24.1%)	0.001*
Hypertension			
Yes	758 (79.5%)	196 (20.5%)	
No	30 (45.5%)	36 (54.5%)	0.0001*
Congestive heart failure			
Yes	162 (89%)	20 (11%)	
No	626 (74.7%)	212 (25.3%)	0.0001*
Coronary artery disease			
Yes	246 (87.5%)	35 (12.5%)	
No	542 (73.3%)	197 (26.7%)	0.0001*
Peripheral vascular disease			
Yes	82 (90.1%)	9 (9.9%)	
No	706 (76.1%)	222 (23.9%)	0.001*
Cerebrovascular Accident			
Yes	55 (84.6%)	10 (15.4%)	
No	733 (76.8%)	221 (23.2%)	0.094

GFR: glomerular filtration rate

BMI: body mass index

CKD: chronic kidney disease

DM: diabetes mellitus

HTN: hypertension

ACEI: angiotensin-converting enzyme inhibitors

ARBs: angiotensin receptor blockers

**Table 4:** Degree of eGFR decline among patients with progressive CKD

Factors	Degree of eGFR decline ml/min/1.73 m^2^/year	P value
**Cause of CKD:**		
DM and HTN	3.2 ± 4.3*	
DM	2.4 ± 2.0	
HTN	2.2 ± 1.8	
Others	2.3 ± 2.5	0.001
**Albuminuria, at time of diagnosis:**		
Grade:		
A1	1.6 ± 1.5*	
A2	2.7 ± 4.4*	
A3	3.2 ± 2.4	0.0001
Hemoglobin A1c %, at time of diagnosis		
< = 6	2.2 ± 2.1	
>6 and < = 8	2.4 ± 2.0	
>8 and < = 10	2.8 ± 2.0	
>10	4.1 ± 6.2*	0.0001
Use of ACEI/ARBs		
Yes	2.3 ± 2.9	
No	3.3 ± 3.7	0.0001

GFR: glomerular filtration rate

CKD: chronic kidney disease

DM: diabetes mellitus

HTN: hypertension

ACEI: angiotensin-converting enzyme inhibitors

ARBs: angiotensin receptor blockers
